# Nanoscale Differences in the Shape and Size of X and Y Chromosome-Bearing Bovine Sperm Heads Assessed by Atomic Force Microscopy

**DOI:** 10.1371/journal.pone.0059387

**Published:** 2013-03-19

**Authors:** José O. Carvalho, Luciano P. Silva, Roberto Sartori, Margot A. N. Dode

**Affiliations:** 1 Laboratory of Animal Reproduction, Embrapa Genetic Resources and Biotechnology, Brasilia, Brazil; 2 Laboratory of Mass Spectrometry, Embrapa Genetic Resources and Biotechnology, Brasilia, Brazil; 3 Department of Animal Science, University of São Paulo, Piracicaba, Brazil; 4 School of Agriculture and Veterinary, University of Brasília, Brasília, Brazil; Dalhousie University, Canada

## Abstract

Sperm dimensions and the question of whether X and Y chromosome-bearing sperm differ in size or shape has been of great interest, especially for the development of alternative methods to sort or classify sperm cells. The aim of the present study was to evaluate possible differences in the shape and size of the sperm head between X and Y chromosome-bearing sperm by atomic force microscopy (AFM). One ejaculate per bull (n = 4) was used. Each ejaculate was separated into four fractions: non-sexed (NS), sexed for X-sperm (SX), sexed for Y-sperm (SY) and a pooling of SX and SY samples (SXY). Using AFM, 400 sperm heads per group were measured. Twenty three structural features were assessed including one-, two- and three-dimensional parameters and shape descriptors. These measurements determine the micro- to nanoscale features of X- and Y-bearing chromosomes in sperm cells. No differences were observed for any individual variables between SX and SY groups. Next, a simultaneous evaluation of all features using statistical discriminant analysis was performed to determine if it was possible to distinguish to which group belong each individual cells. This analysis clearly showed, a distinct separation of NS, SXY, SX and SY groups. The recognition of this structural possibility to distinguish between X and Y sperm cell might improve the understanding of sperm cells biology. These results indicated that the associations of several structural measurements of the sperm cell head are promising candidates for development of a new method of sperm sexing.

## Introduction

Since the discovery by Painter [1] that sperm cells carry either an X or Y chromosome, there has been continuing interest in technologies that enable sperm sorting into X and Y chromosome-bearing fractions. Sperm sorting can have a great impact on breeding programs and has considerable economic value in the livestock industry. Additionally, sperm sorting can be used for human applications where such a technique is important in the prevention of sex related diseases [2].

Although several methods have been developed for sperm sex determination, the only method effective for routine use is ﬂuorescence-activated cell sorting using ﬂow cytometry [3]. Sorting by flow cytometry is based on differences in the DNA content of X and Y chromosome-bearing sperm cells; the differences in X and Y sperm is approximately 4% in bovine [4]. However, the reduced pregnancy rates or embryo production in artificial insemination (AI) and embryo transfer (ET) programs using sexed sperm by flow cytometry [5]–[11] has motivated the search for alternative methods. The development of new sorting methods could eliminate the potential hazards that result from the flow cytometry technique, which requires DNA staining and UV excitation.

However, to develop new approaches for sexing sperm, it is crucial to identify one or more characteristic parameter other than DNA content that successfully distinguishes X from Y chromosome-bearing cells. Some studies have revealed differences in the head volume of X- and Y-bearing bovine sperm [2]. Similarly, the human sperm heads of the X chromosome-bearing sperm cells are larger and longer than Y chromosome-bearing sperm [12], [13]. These results indicate that the potential differences in size or shape of X- and Y-bearing sperm heads are potential candidates for criterion to separate the two sperm populations. Nevertheless, a study by Zavaczki et al. [14], in which more than 2,000 human sperm cells were assessed has raised doubts about whether there is any real variation between X and Y chromosome-bearing sperm cells.

In the studies in which no variation was observed between X and Y sperm cells, sperm were photographed using a light microscope and their structural dimensions were manually determined 2,12,13. Therefore, the possible errors and limitations of the technique could be responsible for the lack of consistent differences in the results. In fact, the finer details of sperm substructure cannot be detected by these manual methods, which offer limited resolution and require elaborate sample preparations [15]. Therefore, there are no accurate data regarding the real magnitude of the structural differences between X- and Y-bearing sperm. Independent measurements using other high-resolution techniques could provide new information for determining the differences between X and Y sperm. In this respect, atomic force microscopy (AFM) has opened up new possibilities to investigate the structure of biological materials such as sperm cells. AFM is a type of high-resolution scanning probe microscopy mainly used to map the topographic surface of samples [16]. When applied to the study of cell morphology, AFM gives detailed three-dimensional information on cell biophysics and structure and provides insight into both the physiological and pathological changes in the cells. Additionally, AFM provides imaging of the surface of cells, at nanometer resolution, almost in real time [17]. Therefore, AFM has been used to determine structural and topological features of bovine, human and rabbit spermatozoa for morphological analysis of the acrosome-intact and -reacted, as well as for structural changes during maturation and capacitation [15], [18]–[22]. However, no studies have used AFM to evaluate some potential variations in the measurements of the head of X- or -Y spermatozoa.

Therefore, the objective of this study was to evaluate potential differences in the shape of the head between X and Y chromosome-bearing sperm by AFM. We found that when a simultaneous evaluation of all the features assessed by AFM in a statistical discriminant analysis was used, it was possible to clearly distinguish the X- and Y-bearing sperm. This knowledge will improve the understanding of sperm biology and can be used for the development of new methods for sperm sexing.

## Materials and Methods

### Separation of X and Y Chromosome-bearing Sperm

To obtain X and Y sperm cells sorting using flow cytometry, semen obtained from four sexually mature Nellore bulls was used. One ejaculate from each bull was collected using an artificial vagina, and only ejaculates with ≥60% motility and ≤20% morphological abnormalities were used. Each ejaculate was divided into three fractions. One fraction was used as the non-sexed (NS) semen, and the other two were submitted to flow cytometry and sorted for X (SX) and Y (SY) chromosome-bearing fractions. The proportion of semen designated for immediate freezing was diluted in Tris-base freezing diluent with 4% egg yolk, cooled at 4°C for 90 min and then diluted with Bioxcell® (IMV, L’Aigle, France). Sperm were loaded into 0.5 mL straws (IMV, L’Aigle, France) and frozen in a programmable freezer, TK 3000® (TK, Uberaba, MG, Brazil). At the end of the program, the straws were submerged in nitrogen for storage.

The remainder of the ejaculate was diluted to 200×10^6^ sperm/mL with Tris medium supplemented with 49 to 65 mM Hoechst 33342 (Invitrogen Molecular Probes®, Eugene, OR, USA) and incubated for 45 min at 35°C. After staining, samples were diluted 1∶1 with Tris medium supplemented with 4% egg yolk and 0.0015% food dye (FD&C #40; Warner Jenkinson Company Inc.®, St. Louis, MO, USA) and filtered through a 50 µm filter (GCAT, Fort Collins, CO, USA) to remove any debris or agglutinated cells prior to sorting.

A high-speed cell sorter (MoFlo SX, Beckman Counter, CA, USA**)** was operated at 40 psi with a diode pumped solid-state pulse laser (Vanguard 350 HMD-355; Spectra Physics, Mountain View, CA, USA) at 125 mW with bovine sheath fluid (CHATA Biosystems Inc.®, Fort Collins, CO, USA). Gates were set to attain 90% purity, and the sexed sperm were sorted into Tris I medium. After being cooled at 4°C for 90 min, the sexed sperm were centrifuged and diluted into Bioxcell® (IMV, L’Aigle, France). The semen was packaged into 0.25 mL straws and frozen as described above for non-sexed sperm.

All the semen collection, sorting and frozen was done by commercial company (ABS Pecplan®, Uberaba, Brazil).

### Sperm Processing for Analysis

One straw per group (SX and SY) for each bull was thawed and a fourth group was formed by pooling SX and SY samples (SXY). After the straws were thawed, one aliquot per group was removed to assess acrosome integrity using fluorescent probe isothiocyanate-conjugated peanut agglutinin (FITC-PNA) and propidium iodide (PI), as previously described [23]. Briefly, an aliquot (10 µL) of a thawed sperm sample was diluted with staining solution (30 µL) and incubated for 10 min. The staining solution consisted of buffered formal saline, sodium citrate (3%), PI (0.75 mM), and FITC-PNA solution (1 mg/mL in PBS). An aliquot (5 µL) of stained suspension was placed on a slide and covered with a coverslip. At least 200 sperm were examined under a phase contrast and epifluorescence microscope (1000×; Axiophot Zeiss®; barrier filter 484/518 nm excitation/emission). Sperm labeled in red with PI were considered alive. Living cells were classified as acrosome-reacted, if the acrosome had uniform FITC-PNA green fluorescence, or as acrosome-intact, if no fluorescence was visible. The remaining sperm sample was centrifuged for 5 min at 200×g to isolate sperm cells from the extender. The supernatant was discarded and the pellet was fixed for 5 min in 1 mL of formol saline (1.6%) and then centrifuged for 5 min at 200×g. After centrifugation, the supernatant was discarded and the pellet was washed by centrifugation twice with 1 mL of sterile water for 5 min at 200×g. The pellet was resuspended in 30–200 µL sterile water. Finally, 2 µL aliquots of sperm suspension were put on glass coverslips and air dried for assessment within atomic force microscopy.

### Atomic Force Microscopy Analysis

AFM analysis was performed on sperm cells in air using the SPM-9600 equipment (Shimadzu, Japan) as described by Bonatto et al. [24]. The images were acquired in constant force contact mode using 200 µm-length V-shaped cantilevers (nominal spring constant of ∼0.15 N/m, resonant frequency of 24 kHz) with integrated pyramidal tips (curvature radius <20 nm). The scanner has a range of 125 µm in XY-directions and 7 µm in the Z-direction. All AFM images were acquired as 512×512 pixels at a scan rate of 1 Hz. The images were processed using SPM-9600 off-line software. The processing consisted of an automatic plane fit leveling of the surface. One hundred individual cells per animal for each group were manually segmented using digital zoom of the original image using the labeling function of the particle analysis software. Then, cell measurements were performed on the sperm head. Twenty three characteristics were assessed, including shape descriptors and one-, two- and three-dimensional parameters. The shape descriptors were obtained using a mathematical formula with one- and two-dimensional values (for details, see mathematical formulas below).

### Mathematical Formulas used to Generate the Shape Descriptors

Form factor: (4*pi*Area excluding hole)/(Perimeter*Perimeter).

Roundness: (4*Area including hole)/pi*(Maximum diameter*Maximum diameter).

Aspect ratio: Maximum diameter/Pattern width.

Effective diameter: (Area including hole/pi)*2.

Distortion: this shape descriptors was done according manufacturer.

Circular degree: pi*maximum diameter/4*area excluding hole.

Circularity ratio: (4*pi*Area including hole)/Perimeter*Perimeter.

Thin degree: maximum diameter/pattern width.

Compact aspect rate: (square root((4/pi)*Area including hole))/Maximum diameter.

Elongation: (Perimeter*Perimeter)/Area including hole.

Roughness: (perimeter*perimeter)/(4*pi*area excluding hole).

Degree of circularity: (2*square root(pi*Area including hole)))/Perimeter.

### Statistical Analyses

A total of 23 characters were analyzed with the contrast media using the theory of generalized linear models (PROC GLIMMIX; SAS 9.1, SAS Inst. Inc., Cary, NC, USA). The means of the characters were compared among groups by F-test at a significance level of α = 0.05. Data are presented as means ± SEM. The performance of the 23 characters was also collectively evaluated by discriminant analysis (complete estimation and tolerance of 10^−20^) in respect to sexing prediction.

## Results

Because the absence of acrosome can affect the volume of the sperm head, we evaluated the acrosome status in all samples used. No differences in the percentage of the cells with intact acrosome were detected among NS (86.6±3.3%), SXY (87.2±3.3%), SX (86.0±3.3%) and SY (89.4±3.3%) groups.

**Figure 1:**
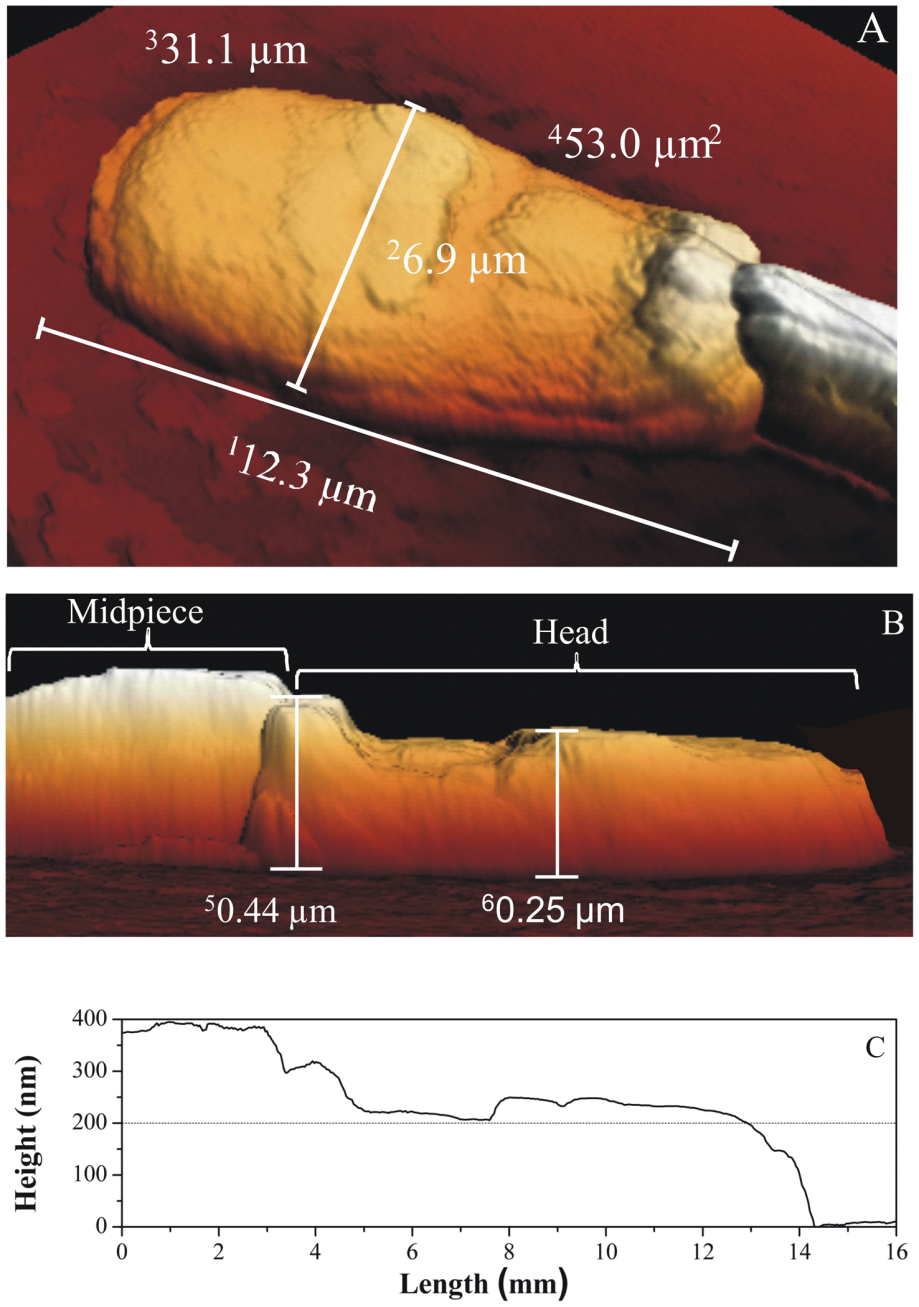
Atomic force microscopy (AFM) 3D view images (A and B) and line profile (C), showing different dimensional parameters of the bovine sperm cells containing an X-chromosome. 1. Maximum diameter; 2. Width; 3. Perimeter; 4. Surface area; 5. Maximum height; 6. Average height.

For the AFM analysis, 23 structural characteristics were assessed in sexed and non-sexed sperm. The results are presented in Tables 1, 2 and 3. These measurements determine the features of X- (Figure 1 A–C) and Y-bearing chromosomes in *Bos indicus* sperm. When SX and SY groups were compared, no differences were observed for any individual variables (Table 1, 2 and 3). However, the NS group presented a higher minimum height, elongation and membrane roughness and a lower form factor, circularity ratio and degree of circularity than the SXY group (Tables 1 and 3).

**Table 1:** Values (± SE) of one-dimensional measurements of the non-sexed (NS), pool of the sorted bovine sperm cell for X and Y (SXY), sorted for X (SX) and sorted for Y (SY).

Groups	Measures of one dimensional structural characteristics						
	Means Radius (µm)	Means radius variance (µm)	Maximum height (µm)	Minimum Height (µm)	Average height (µm)	Maximum diameter (µm)	Pattern width (µm)
**NS**	3.99±0.21	1.02±0.05	0.47±0.03	0.15±0.02^a^	0.29±0.02	11.97±0.59	7.14±0.38
**SXY**	3.99±0.08	1.01±0.06	0.45±0.01	0.13±0.01^b^	0.28±0.01	10.94±0.36	7.46±0.31
**SX**	4.09±0.32	1.02±0.06	0.46±0.02	0.14±0.01	0.29±0.02	11.19±0.89	7.42±0.98
**SY**	4.08±0.22	1.02±0.07	0.48±0.07	0.14±0.02	0.30±0.04	11.15±0.66	7.70±0.17

a,b Within each column, differences between NS and SXY group (P≤0.05).

Values are an average of at least 400 sperm for each group.

**Table 2:** Percentage (± SE) of two- and three-dimensional measurements of the non-sexed (NS), pool of the sorted for X and Y (SXY), X-bearing sperm (SX) and Y-bearing bovine sperm (SY).

Groups	Measures of structural characteristics				
	Two dimensional			Three dimensional	
	Perimeter (µm)	Area including hole (µm^2^)	Surface area (µm^2^)	Volume (µm^3^)	
**NS**	28.4±1.55	46.4±5.0	46.9±4.97	13.7±1.44	
**SXY**	28.3±0.65	46.5±5.4	47.1±1.66	13.2±0.65	
**SX**	29.9±2.25	49.0±7.7	49.6±7.68	14.4±1.71	
**SY**	28.9±1.59	48.9±5.3	49.4±5.32	14.9±2.68	

Values are an average of at least 400 sperm for each group. There was no difference between groups (P>0.05).

**Table 3:** Values (± SE) of the shape descriptors parameters of the non-sexed (NS), pool of the sorted for X and Y (SXY), X-bearing sperm (SX) and Y-bearing bovine sperm (SY).

Groups	Form factor	Roundness	Aspect ratio	Effective diameter	Distortion	Circular degree	Circularityratio	Thin degree	Compactaspect rate	Elongation	Roughness	Degree of circularity
**NS**	0.71±0.01^a^	0.49±0.01	1.65±0.03	7.67±0.41	0.71±0.02	2.05±0.01	0.72±0.01^a^	1.65±0.03	0.70±0.01	17.50±0.02^a^	1.39±0.01^a^	0.85±0.01^a^
**SXY**	0.72±0.01^b^	0.49±0.02	1.59±0.06	7.69±0.13	0.73±0.01	2.03±0.08	0.73±0.01^b^	1.59±0.06	0.70±0.01	17.30±0.30^b^	1.37±0.02^b^	0.86±0.01^b^
**SX**	0.73±0.01	0.50±0.01	1.64±0.09	7.88±0.60	0.73±0.04	2.02±0.03	0.73±0.01	1.63±0.09	0.70±0.01	17.23±0.07	1.37±0.01	0.85±0.01
**SY**	0.73±0.01	0.50±0.01	1.56±0.06	7.87±0.42	0.73±0.02	2.01±0.04	0.73±0.01	1.56±0.06	0.70±0.01	17.24±0.21	1.37±0.01	0.85±0.01

a,b Within each column, differences between NS and SXY groups (P≤0.05).

Values are an average of at least 400 sperm for each group.

**Figure 2:**
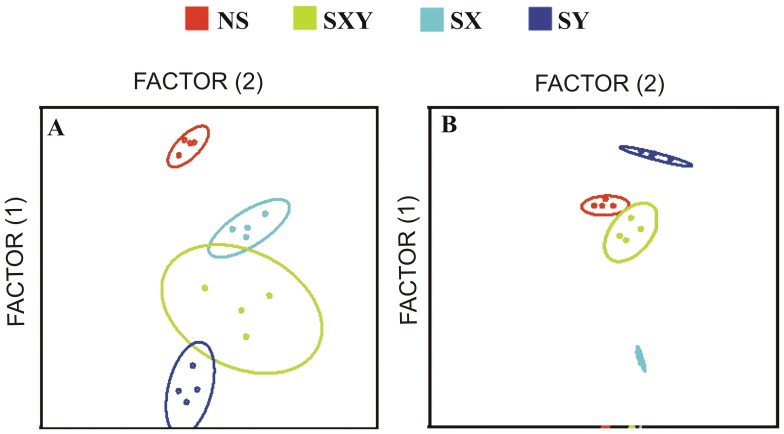
Discriminant analysis of the non-sexed (NS), pool of the sorted for X and Y (SXY), X-bearing sperm (SX) and Y-bearing sperm (SY) using one-, two- and three-dimensional measurements (A) and shape descriptors parameters (B). Each point represents one bull of each group, from the average of 100 cells per bull. The analyses were performed using at least 100 sperm for each bull and 400 sperm for each group.

A simultaneous evaluation of all the measured features by discriminant analysis was performed to determine if it was possible to distinguish to which group belong each individual cells. The results depicted in Figure 2, in which the groups are represented by ellipses of different colors, showed that it was possible to differentiate the different experimental groups NS, SXY, SX and SY with 100% accuracy. Note that despite all the groups were correctly assessed as average parameter values of each sample, not all individual cells clustered within correct groups and this fact can be related to the typical sexing rate error of 10% (data not shown). Discriminant analysis using shape descriptors parameters clearly showed a distinct separation between SX and SY groups (Figure 2 B). Conversely, a more evident separation between NS and SXY was observed when one-, two- and three-dimensional measurements were used in the discriminant analysis (Figure 2 A).

## Discussion

The question of whether X- and Y-bearing spermatozoa differ in size or shape has been of great interest, especially for the development of alternative methods to sort sperm cells. Some reports concerning potential differences [2], [12], [13] between X and Y sperm cells have not revealed distinguishing characteristics [14].

However, in the previous studies, the method used to determine sperm dimensions had limited resolution; the finer details of the sperm could not to be detected or the sample preparation required greater manipulation of the cells. The limitations of the previous methods could be overcome by a more powerful technique for this type of evaluation. AFM has opened up new possibilities to study untreated sperm cells with nanometer resolution and obtain topographic data images of the surface and three-dimensional images of the cells. Therefore, this is the first study using AFM to address potential differences in the size and shape attributes of sperm heads in X- and Y-bearing spermatozoa.

Studies using AFM images identified differences between sperm with intact acrosome and reacted acrosome [20], [21]. The area of the anterior portion of the sperm head with reacted acrosome was approximately 40% smaller than that of sperm with intact acrosome [21]. Because the absence of an acrosome can affect structural characteristics of the sperm cell head, we evaluated the acrosome status of all samples. In the present study, no differences in percentage of cells presenting intact acrosome were detected among the groups. Therefore, the differences found among groups in sperm cell head dimensions could not be related to the presence of the acrosome.

There were no differences in size or shape between SX and SY groups when 23 structural measurements of the sperm cell head were individually analyzed (Tables 1, 2 and 3). Similar results were obtained by Zavaczki et al. [14] using photography taken under phase contrast or differential interference contrast light microscopy. In contrast, differences between X and Y chromosome-bearing sperm in length, perimeter, area and volume have been described in bovine [2] and human spermatozoa [12], [13]. In the previous studies, the authors proposed that the 4% variation in DNA content between X- and Y-bearing sperm cell could lead to the observed differences in volume. In our view, the difference in DNA content is not large enough to be observed as a difference in sperm head volume, which is supported by the results obtained by Zavaczki et al. [14] in which no differences in sperm head dimensions were observed between sperm with XX, XY and YY disomies.

Therefore, differences observed between our study and previous studies may be due to differences in the techniques used. Assessment of the sperm cell by AFM provides topographic images, which can be viewed in 3D, allowing the observation of nanoscale details [15]. Moreover, AFM slides are prepared quickly and easily and under near-physiological conditions [25]. Although AFM imaging can be performed on living cells, a structural/temporal barrier prevents its use with live sperm cell due to the time spent to capture images from one single sample (∼10 min). The sperm is a very dynamic cell and over time it undergoes changes in the plasma membrane caused by membrane destabilization, capacitation and acrosome reaction. These changes could modify the shape of the sperm head during the assessment, making impossible to use intact cells for this type of analysis. To avoid the effect of time on the sperm shape we choose to chemically fix the cells because this procedure can keep the cells in the same stage along the time required for image acquisition. Moreover, since the same conditions for sperm preparation were used for all slides, we can assume that differences between groups may not be due to artifact or distortion caused by sample preparation.

In previous studies, sperm dimensions were based on manual estimation of photography by microscopic images [2], [12], [13]. Additionally, greater manipulation of the sperm was required for sample preparation, including use of dyes or sonication, which could lead to a change in the sperm shape. This hypothesis is based on the fact that when sperm are sonicated to remove the tail, the heads of different sperm break from the tail at different points. The tail contributes to the total sperm-head volume and could increase observed variations in volume.

In an attempt to differentiate X and Y sperm cells, a simultaneous evaluation of all measurements using discriminant analysis was performed. The main purpose of a discriminant analysis is to predict in witch group belong witch, based on a linear combination of the interval variables. The procedure is possible when a set of identical measurements is done in both groups. The end result is a model that allows prediction of to which cell belongs to each group, even though no differences were identified in the individual analysis.

Using all the one-, two- and three-dimensional measurements, it was possible to distinguish averages of all groups with 100% accuracy. However, a clearer separation of the SX and SY groups was obtained when shape descriptor parameters were used for the analysis. Shape descriptors are generated using a mathematical formula with one- and two-dimensional values. This approach could provide greater accuracy in distinguishing the X- and Y-bearing sperm. Our results showed that shape descriptors have an important role in determining the shape of X- and Y-bearing sperm. Therefore, shape descriptors are candidate parameters to be used in new approaches to sexing sperm cells. The knowledge about the almost imperceptible differences in morphology associated with the emerging nanotechnologies will have, through the use of nanoparticles or nanomolecules, extensive application in the field of sperm biology such as in male contraceptive and sperm sexing.

Moreover, the differences in shape descriptors found in our study may be responsible for the variations in movement patterns, including mean angular displacement, linearity and straightness, which can identify differences between X and Y-sperm using computer-assisted sperm analysis [26]. Although sperm movement is initiated at the midpiece and despite the observation that sperm move in response to the flagellum, differences in the shape of the head could influence the hydrodynamics of the sperm head, which can affect sperm movement.

Because flow cytometry is the only proven method for sexing sperm, we used this technique to separate SX and SY sperm prior our investigations. Therefore, to evaluate the effect of the sexing process on the shape of the sperm head, we also compared non-sexed sperm and a pool of X and Y sperm using the same measurements chosen for SX and SY groups. Our results showed for the first time, the effect of sorting process in some measurements of the shape sperm head, such as minimum height, form factor, circularity rate, elongation, roughness and degree of circularity of the sperm (Tables 1 and 3). Because the SX and SY groups were simultaneously submitted to the same process of separation and were obtained from the same ejaculate, we propose that alterations induced by the sexing process had a similar effect on SX and SY groups. This similarity is reinforced by the fact that we did not find any differences in the 23 individual parameters between SX and SY groups.

We speculate that the differences between NS and SXY groups can be related to modifications in the plasma cell membrane. Sperm plasma membrane is composed of protein, phospholipid, cholesterol and other components [27]. It is well known that during the capacitation proteins are removed or redistributed among different regions of the plasma membrane, which modifies its architecture [27], [28]. There is evidence that the sexing process can induce premature capacitation [29] due to membrane destabilization, which could be responsible for the differences in modification of the plasma membrane found between sexed and non-sexed sperm. Indeed, we found a difference in plasma membrane roughness between non sexed and sexed sperm, which could also be related to the premature capacitation reported by others [29].

In summary, we have examined the potential differences between spermatozoa with X or Y chromosomes. Although we did not find differences for any individual variables, by grouping all the structural characteristics evaluated we could distinguish means of population of X- and Y-bearing sperm with 100% accuracy. The clear separation between X and Y sperm was possible due to the high precision of the equipment associated with the discriminant analysis. The recognition of this basic difference between X and Y sperm may improve the understanding of sperm biology. This knowledge can be used as a theoretical basis for the development of alternative methods for sperm sexing.
